# Dacryoendoscopy-assisted incision of Hasner’s valve under nasoendoscopy for membranous congenital nasolacrimal duct obstruction after probing failure: a retrospective study

**DOI:** 10.1186/s12886-021-01948-w

**Published:** 2021-04-19

**Authors:** Yue Li, Min Wei, Xueru Liu, Leilei Zhang, Xuefei Song, Caiwen Xiao

**Affiliations:** 1 Department of Ophthalmology, Shanghai Ninth People’s Hospital, Shanghai Jiao Tong University School of Medicine, Shanghai, China; 2 Shanghai Key Laboratory of Orbital Diseases and Ocular Oncology, Shanghai, China; 3 Handan Eye Hospital, Handan City, Hebei Province China

**Keywords:** Dacryoendoscopy, Nasoendoscopy, Membranous congenital nasolacrimal duct obstruction, probing failure, incision of Hasner’s valve

## Abstract

**Background:**

To introduce a treatment option: dacryoendoscopy-assisted incision of Hasner’s valve under nasoendoscopy and assess its efficacy in treating membranous congenital nasolacrimal duct obstruction (CNLDO) in children older than 1 year with history of initial probing failure.

**Methods:**

52 eyes of 43 children with membranous CNLDO who underwent dacryoendoscopy-assisted incision of Hasner’s valve under nasoendoscopy between May 2012 and October 2020 were enrolled in this retrospective study. All participants were older than 1 year and all the eyes had gone through probing at least once but still had symptoms of epiphora and discharge. Surgical success was defined as a normal fluorescein dye disappearance test (FDDT) and the absence of pre-operation complaints, including epiphora, muco-purulent discharge, increased tear lake or the symptoms of acute infection such as acute dacryocystitis. Patients’ demographics, clinical features and follow-up outcomes were evaluated.

**Results:**

Of all these operated cases, surgical success was achieved in 52/52 eyes without any early or late complications. The overall success rate was 100%.

**Conclusions:**

Dacryoendoscopy-assisted incision of Hasner’s valve under nasoendoscopy is an effective and minimally invasive surgical treatment in membranous CNLDO patients with initial probing failure.

## Background

Congenital nasolacrimal duct obstruction is a common disorder leading to persistent tearing in up to 20% of new-born infants [1–3]. The most common cause of CNLDO is a mucous membrane obstruction at the lower end of the nasolacrimal duct [4–6]. Due to the high percentage of spontaneous resolution for membranous CNLDO (ranging from 32 to 95%) within the first year, most doctors adopt the strategy of “wait and see” or lacrimal sac massage [7–9]. When conservative therapy can’t reach a satisfactory outcome or obstruction persists, probing is recommended as a standard first-line interventional therapy [5, 10]. Even though probing carries a high success rate of 78–97%, there is still a small amount of cases that experience persistent symptoms such as epiphora and mucus discharge after several attempts at probing [11–13]. As a blind and invasive treatment, probing is not free from complications such as bleeding, damage of the nasolacrimal system and of the adjacent structures and inflammation with subsequent nasolacrimal duct (NLD) fibrosis [7, 14]. Damage to the lacrimal duct and subsequent adhesion lead to obstruction again and symptoms recurrent. Further treatment is necessary for these people. Options such as repeated probing or silicon intubation can be chosen. However, repeated probing and intubation also have complications of false passage formation and traumatic obstruction. Other techniques such as balloon catheter dilation and dacryocystorhinostomy (DCR) are more complicated, invasive and controversial in first intention once probing fails [15]. A safer surgical approach with a lower rate of complications and a high success rate is in demand. Here we introduce a technique which combines dacryoendoscopy and incision of Hasner’s valve under nasoendoscopy as a less invasive and more reliable procedure. After years of treating patients who had suffered previous probing failure, we evaluated in this retrospective study the safety and effectiveness of this procedure in 43 consecutive membranous CNLDO children.

## Materials and methods

**Fig. 1:**
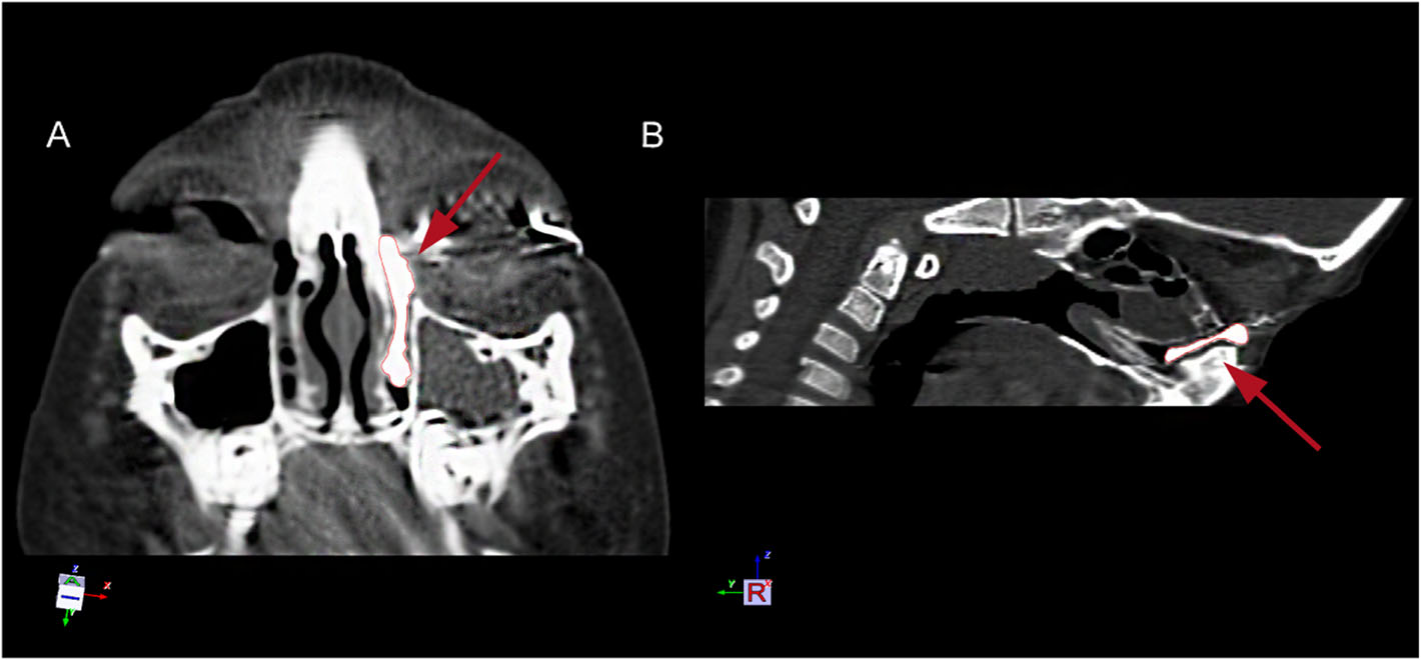
CT-DCG of a membranous CNLDO patient. **a**. An axial image. **b**. A sagittal image. **a** and **b**. The contrast medium was blocked at the end of the nasolacrimal duct, showing the shape of the whole duct. The enlarged Hasner’s valve was shown by the red arrows

This is a retrospective study of all children with membranous CNLDO who had failed probing and then underwent dacryoendoscopy-assisted incision of Hasner’s valve under nasoendoscopy between December 2012 and September 2020. This study adhered to the principles of the Helsinki Declaration and was approved by the Ethics Committee of Shanghai Ninth People’s Hospital. Only membranous CNLDO patients ≥1 year old and had previously failed probing were included in this study. The diagnosis of membranous CNLDO was based on the history of epiphora, mucopurulent crusting and evaluation of the irrigation tests. CT dacryocystography (CT-DCG) was done additionally if the diagnosis of a membranous obstruction was uncertain (Fig. 1). CT-DCG was also used to exclude bony nasolacrimal duct stenosis and bone atresia. Children with a genetic syndrome, punctual anomalies, a history of trauma to the nasolacrimal system, and/or craniofacial abnormalities were also excluded. All the eyes with a membranous obstruction underwent dacryoendoscopy-assisted incision of Hasner’s valve under nasoendoscopy. Surgical success was defined when the following post-operative conditions were satisfied: no epiphora, normal tear meniscus height, and a normal FDDT. All patients had a 3 month follow-up period. The following clinical data were collected from patients’ medical records: sex, age, laterality, times of previous probing, videos of dacryoendoscopy and nasoendoscopy during operation and surgical success.

**Fig. 2:**
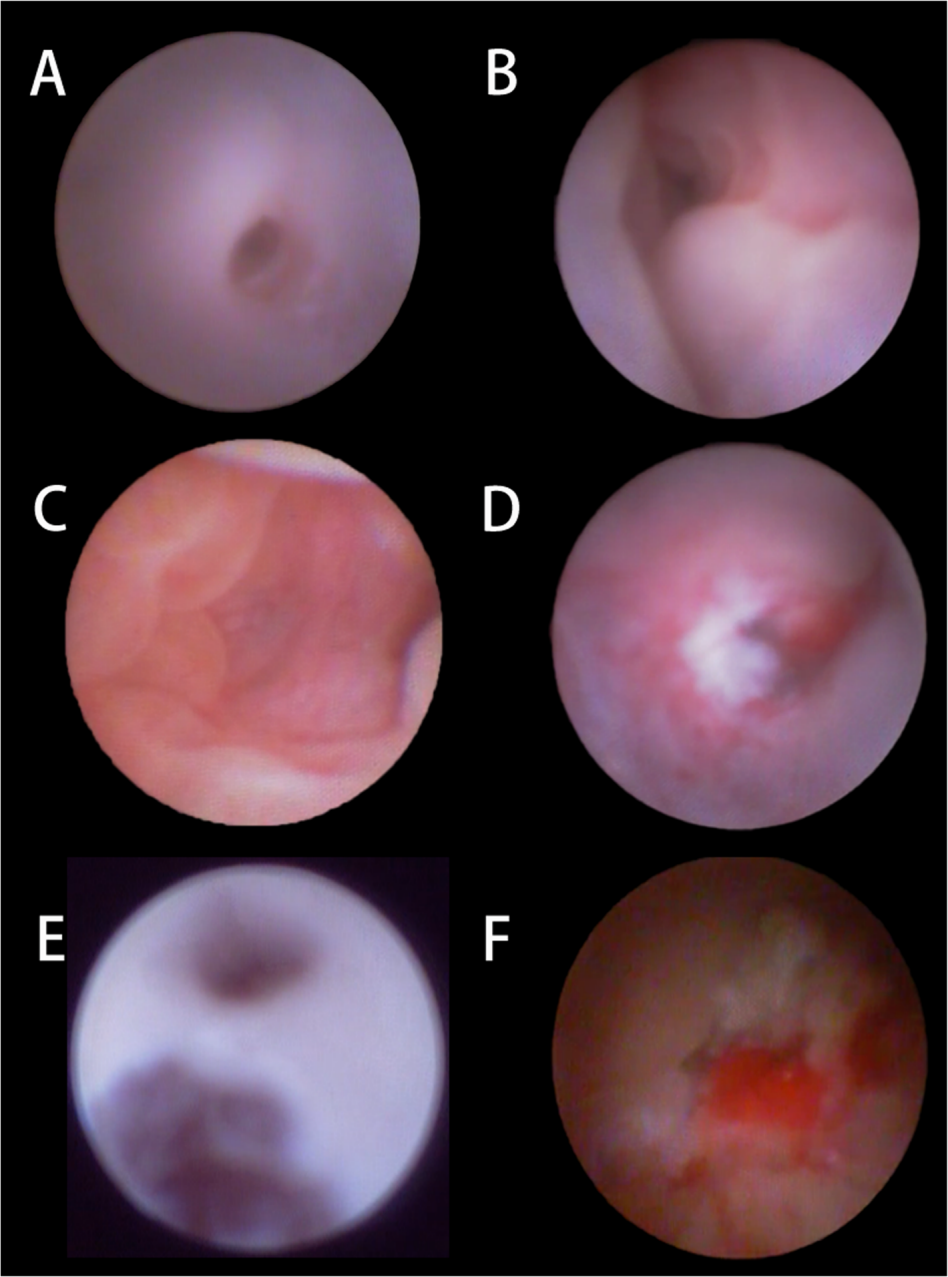
Dacryoendoscopic images of membranous CNLDO patients during operation. **a**. The normal lacrimal canaliculus. **b**. The surrounding area of the common canalicula. The valve of Rosenmüller was visible. **c**. The obstruction point- the distal end of the NLD. **d**. An irregular scar between the lacrimal sac and the lacrimal canaliculus. **e**. The false passage near the common canaliculus. **f**. Inflammation and secretions inside the lacrimal duct

**Fig. 3:**
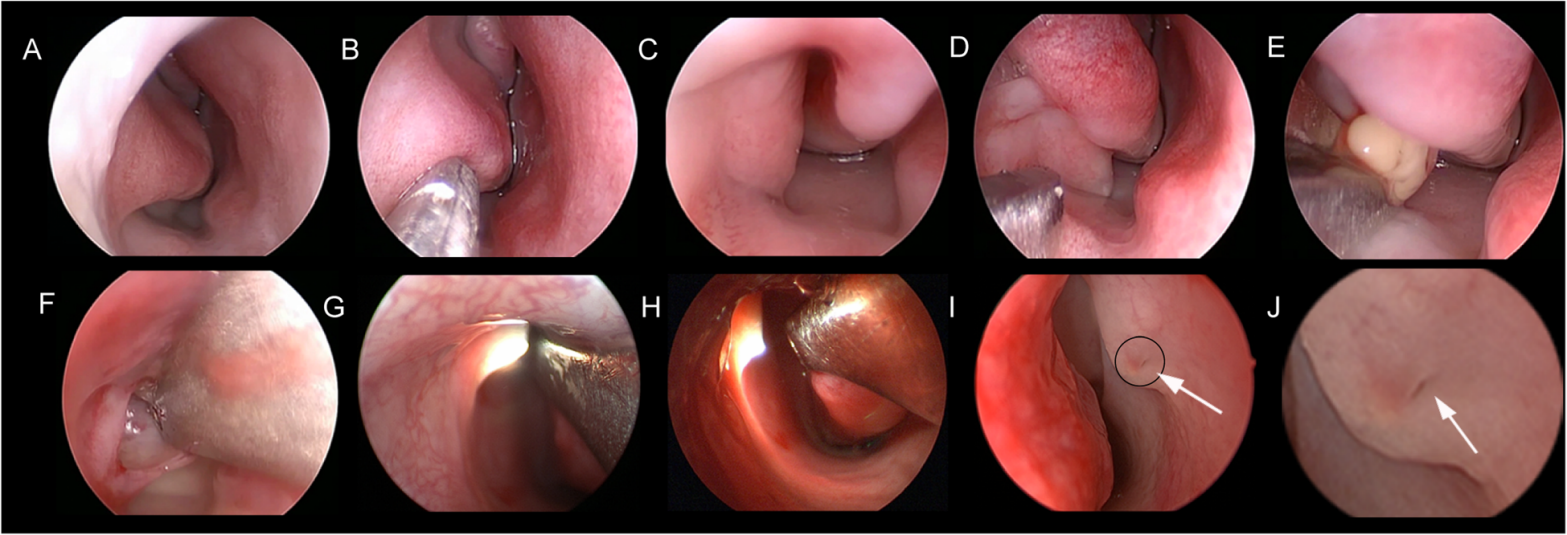
Nasal endoscopic images during operation. **a**. The endoscopic finding of the nasal cavity. The abnormal Hasner’s valve was covered by inferior turbinate. **b**. The inferior turbinate was pushed towards the nasal cavity to widen the surgical space. **c**. The end of NLD was thick and overdeveloped. **d**. A Crawford probe was inserted very gently into the distal end of the nasolacrimal duct and the probe and surrounding mucosa protruded shaped like a tent. **e**. The membrane at the distal end of the NLD was fully incised using a sickle knife; after that the pus and secretions rapidly flowed into the inferior duct. **f**. The surplus membrane in the distal end was separated and cut off using superfine mucosal scissors. **g & h**. Light from dacryoendoscope was used to identify the exact position of end of the NLD from the vision of nasal endoscopy. **i**. Previous probing left a very tiny hole (the arrow) at the mucosa of the abnormal Hasner’s valve but the lacrimal duct was not fully opened. **j**. Magnification of the image inside the black ring in Fig. 3i.

Surgical technique: All surgeries were performed by a single clinician under general anaesthesia. The lacrimal puncta was infiltrated with Oxybuprocaine Hydrochloride Eye Drop. After anaesthesia, the upper punctum was dilated using a punctual dilator and a dacryoendoscope (Polydiagnost GmbH, Freiburg Germany) was inserted very tightly. Mucosa of whole lacrimal duct and the obstruction point (usually the failed canalization distal end of the NLD) was observed through the endoscopic passages (Fig. 2a-f). This was followed by the precise placement of two neurosurgical pledgets soaked in xylometazoline hydrochloride-one pledget under the inferior turbinate, the other between the inferior turbinate and nasal septum in order to constrict the vessels of the nasal mucosa for better visualization. After that, the nasal cavity was examined using 0° rigid nasal endoscope with an outside diameter of 2.7 mm (Fig. 3a). Mucosa forceps were used to push the inferior nasal turbinate as well as the lateral nasal wall to widen the surgical space and show the inflated distal end of the nasolacrimal duct (Fig. 3b and c). Assisted with the light transmission from both dacryoendoscope and the nasal endoscopy, location and variable thickness of the membrane of Hasner’s valve were identified (Fig. 3g and h). At this point, we used a Crawford probe fitted onto the dacryoendoscope sheath for probing the blockage. When the Crawford probe reached the distal end of the NLD, a tent-shaped protrusion on the membrane was created, which was obvious enough to identify the distal of NLD through nasoendoscopic vision (Fig. 3d). Then under nasoendoscopy, the membrane at the distal end of the NLD was fully opened using a sickle knife, after which pus and other secretions flowed into the inferior nasal meatus (Fig. 3e). After incision, the dilated membrane was pruned using mucosa cutters or superfine mucosal scissors until there was a fissure at Hasner’s valve wide enough to allow tear and other secretions to flow uninterruptedly into the inferior nasal meatus (Fig. 3f). Intubation was used to prevent re-obstruction caused by post-operation adhesion or edema inside the lacrimal duct. After surgery, budesonide nasal spray was used for 1 week to reduce edema of nasal mucosa. Antibiotic eyedrops levofloxacin were used for 3 days to prevent infection. Silicon tube was removed under local anaesthesia providing patients showed no signs of epiphora and had a normal FDDT, usually around 14 days after operation (14.09 ± 2.00 days). All patients had a 3 month follow-up period.

## Results

Patients data is shown in Table 1. In total, 52 eyes of 43 children were included in this study, which consisted of 31 left eyes and 21 right eyes. The male to female ratio was 20:23. The age of these children ranged from 12 months to 13 years with a mean age of 3.28 years. Times of probing before our surgery ranged from once to as many as 8 times in a 5 year old boy. 32 of these eyes had failed probing once and 20 had failed probing twice or more. After operation, 48 eyes’ symptoms disappeared within 3 days. 4 eyes’ symptoms lasted longer than 3 days but all disappeared within 1 month. The overall success rate was 100% and none of these patients had complications during our 3 month follow-up period. Besides, considering the significant heterogeneity of the patients’ age, we stratified the severity of the disease related to the age of the involved patients based on the total scores of their symptom and pathological presentation under dacryoendoscopy. Score of symptom was based on the most established version-the Munk score, according to which, 0 = no epiphora; 1 = occasional epiphora requiring dabbing with a tissue or handkerchief less than twice a day; 2 = epiphora requiring dabbing 2 to 4 times a day; 3 = epiphora requiring dabbing 5 to 10 times a day; 4 = epiphora requiring dabbing more than 10 times a day or constant tearing [16]. Score of pathological presentation was based on the image of the lacrimal duct under dacryoendoscopy, which was defined as: 0 = normal; 1 = slight edema of lacrimal duct mucosa; 2 = hyperemia of lacrimal duct mucosa, mucus secretions or inflammation inside the duct; 3 = scars including false passage of lacrimal duct. The total scores were the sum of the above two scores, which was defined as 0–2 = mild, 3–5 = moderate, 6–7 = severe. The severity of the disease was separately described in 3 age groups as shown in Table 2. The Kruskal-Wallis test was carried out and *P* = 0.432, showing there was no correlation between age and severity of the disease in our data (statistical significance was considered for *P*-value < 0.05).

**Table 1:** Clinical data of membranous CNLDO patients

Item		Number	Percentage
Gender	Male	20	46.51%
	Female	23	53.49%
Age (years)			
range	1 to 13		
mean (standard deviation)	3.28 (2.86)		
Sides (cases)	Left	22	51.16%
	Right	12	27.91%
	both	9	20.93%
Time of previous probing			
range	1 to 8		
mean (standard deviation)	1.96 (1.53)		
	1	32	61.54%
	≥2	20	38.46%
Surgical success (eyes)	Yes	52	100.00%
	No	0	0.00%
Complications (eyes)	Yes	0	0.00%
	No	52	100.00%

**Table 2:** Severity of membranous CNLDO in different age groups

Age (months)	Severity of membranous CNLDO (eye)			In total (eye)	
	mild	moderate	severe		
12 to 35	1	16	13	30	
36 to 71	0	7	10	17	
72 to 156	1	2	2	5	

It’s worth mentioning that through evaluation of dacryoendoscopy and nasoendoscopy videos during operation we noticed the reasons for patients’ recurrent symptoms after probing. From dacryoendoscopy videos during operation, we found injuries to the lacrimal duct including inflammation, scars, adhesions and false passages in 36 eyes (as shown in Fig. 2d-f). This seemed to explain the re-obstruction of the nasolacrimal duct and was to blame for patients’ recurrent symptoms just as previous studies had reported. However, we noticed in 16 cases the lacrimal duct was not injured without inflammation or exudation or adhesion but still had probing failure. Combined with nasoendoscopy videos of these children, we found the Hasner’ s valve was not perforated at all in 4 eyes while in 12 eyes the nasolacrimal duct was not fully open with just a tiny hole at Hasner’ s valve (Fig. 3i and j) which wouldn’t allow tears and secretion to flow uninterruptedly. In 36 eyes with lacrimal duct mucosa injuries, the tiny hole also existed in 26 eyes of them and 7 eyes weren’t perforated at all. (Table 3).

**Table 3:** Dacryoendoscopy and nasoendoscopy finding of patients during operation (eyes)

Nasoendoscopy	Hasner’s valve fully open	A tiny hole at Hasner’s valve	Hasner’s valve not open	Total
Dacryoendoscopy				
With lacrimal duct mucosa injuries	3 (5.77%)	26 (50.00%)	7 (13.46%)	36 (69.23%)
Without lacrimal duct mucosa injuries	0 (0%)	12 (23.08%)	4 (7.69%)	16 (30.77%)
Total	3 (5.77%)	38 (73.08%)	11 (21.15%)	52

## Discussion

Probing, serving as first-line invasion therapy for membranous CNLDO, is convenient and has a relatively high success rate [17]. However, there were still some children we encountered who had complaints of epiphora and discharge after probing. This showed the failure of their previous probing. With regards to treatment options following a failed initial probing, doctors all over the world haven’t reached a common conclusion. They may choose to carry out a repeated probing or there are some other advanced treatments such as silicon intubation, balloon dacryocystoplasty and DCR [18]. Repeated probing has a lower success rate than initial one, as the risk of probing failure remains and probe may go into the cicatricial structures or false passage an unsuccessful initial probing caused, as shown in our Fig. 2d-f [19]. Silicone intubation can also be adopted, but it has complications including the creation of false passages, erosion or slitting of the punctum, and the formation of pyogenic granulomas [5, 20]. Stenting probing is a procedure reported to have a mean success rate of 41.8–66.5% (CI 95%) for treating NLD in adulthood and can also be applied to treating membranous CNLDO in childhood [21]. Nevertheless, it has complications similar to blind probing and silicone intubation and the success rate decreases as patients’ age increases and is only 45.2% in patients older than 6 years old [22]. DCR is the last resort of the intervention in children with persistent CNLDO including bony obstruction [23, 24]. It’s quite effective but requires high surgical skill and optimal equipment [25]. Besides, with the destruction of bone during operation, DCR is the most invasive option and not recommended in first attempt after probing failure.

Here we used dacryoendoscopy-assisted incision of Hasner’s valve under nasoendoscopy for membranous CNLDO in children who had a history of failed probing, which was safer and less potentially damaging with less chance of complications. In this approach we combined dacryoendoscopy and nasoendoscopy to provide accurate vision and precise incision of the Hasner’s valve, reaching a success rate of 100%. Dacryoendoscopy is a non-invasive method that has been used to directly view, localize and probe the obstructions in the lacrimal drainage system precisely [26]. Nasoendoscopy is used to observe the nasal cavity and conduct some operations. The advantages of their co-operation are as follows. First, during this operation, the dacryoendoscopy can visualize the lacrimal cavity with accuracy. This enables surgeons to arrive directly at Hasner’s valve and perform direct dacryoendoscopic probing of the obstruction point with less chance of causing injury to the mucosa of the lacrimal duct or go into false passage caused by a failed blind probing before (as shown in Fig. 2d-f). Secondly, when the dacryoendoscopy reached the obstruction point, light emitted from it is traceable through the membrane of the Hasner’s valve and can be observed under nasal endoscopic vision (as shown in Fig. 3g and h). This further aids in locating the Hasner’s valve from the nasal side and allows precise position for incision. Third, turning the dacryoendoscopy towards the nasal side to make the membrane protrude results in an easy and minimally invasive incision and pruning of the membrane. Being carried out through endoscopy, this technique is relatively safer and less invasive than most options and won’t cause any scar. Also, through simple incision of Hasner’s valve, it’s a simple procedure easy to learn.

Besides, with the help of a dacryoendoscopy and a nasal endoscopy, we can have a better understanding of the reason for previous probing failure. There are mainly two reasons for probing failure. The first is the more often reported-bleeding, scarring or a false passage which is a common complication of blind probing. The use of a dacryoendoscopy provides direct vision of the lacrimal duct so we won’t injure the lacrimal duct mucosa. And intubation under dacryoendoscopy after incision of the Hasner’s valve can prevent further adhesion of the lacrimal duct and keep the patency. The second is that in some cases, overdevelopment at the distal end of the nasolacrimal duct may cause a thickened membrane which can’t be perforated by traditional simple probing [4, 27]. In these cases a small hole of Hasner’s valve has already formed but not big enough to allow tear drainage when the liquid or secretion inside the lacrimal duct increased (Fig. 3i and j). This explains why some children’s symptoms disappear temporarily after probing but re-emerge soon. Through our procedure the abnormal membrane, the over-development of the NLD distal end or the thickened Hasner’ valve can be incised and a fissure similar to physiological Hasner’s valve and large enough to allow tear and secretions to flow freely in the nose is formed. So our procedure is effective to fully open the abnormal Hasner’s valve with circumvention of injury of the lacrimal duct mucosa and thus doesn’t have complications in traditional probing and intubation.

It is also worth mentioning that given the chance of spontaneous resolution and the risk of complications with general anaesthesia, it is recommended that the surgery be performed in patients older than 1 year. And our procedure is aimed at resolving the membranous obstruction and may not work for bony obstruction. Therefore careful examinations need to be made before surgery.

The limitations of the study include our sample size being not so large and the lack of a randomized controlled trial. The trial will need to be well designed and conducted in the future. Another limitation is that due to dacryoendoscopy as well as nasoendoscopy used in this procedure, the cost is relatively higher than a single endoscope system.

## Conclusions

The approach we share can deal with membranous CNLDO after probing failure with a success rate of 100%. Under vision of dacryoendoscopy and nasal endoscopy, we re-opened Hasner’s valve and reconstructed the patency of the lacrimal duct in an effective and minimally invasive way with less chance of complications. In conclusion, it’s a reliable, effective and safe treatment for membranous CNLDO patients older than 1 year with initial probing failure.

## Data Availability

Most of data generated or analyzed during this study are included in this published article. The remaining datasets are available from the corresponding author upon reasonable request.

## Abbreviations

CNLDO: Congenital nasolacrimal duct obstruction
FDDT: Fluorescein dye disappearance test
NLD: Nasolacrimal duct
DCR: Dacryocystorhinostomy
CT-DCG: Computed Tomography dacryocystography
